# Supplementary material for: Microtubule association induces a Mg-free apo-like ADP pre-release conformation in kinesin-1 that is unaffected by its autoinhibitory tail
Source: Nat Commun. 2025 Jul 5;16:6214. doi: 10.1038/s41467-025-61498-3 (PMC12228791; doi:10.1038/s41467-025-61498-3)
Supplement: Supplementary file 1 — Supplementary Information [file 41467_2025_61498_MOESM1_ESM.pdf]

Supplementary Information for

Microtubule association induces a Mg-free apo-like ADP pre-release conformation in kinesin-1 that is unaffected by its autoinhibitory tail

Atherton J[1,#,*], Chegkazi MS[1,#,⊥], Leusciatti M[2,3], Di Palma M[2], Peirano E[2], Pozzer LS[2], Meli MVA[3], Pasqualato S[4], Foran T[1], Morra, G[3], Steiner RA[1,2,*]

[1]Randall Centre for Cell and Molecular Biophysics, King's College London - New Hunt's House, Guy's Campus, London SE1 1UL, United Kingdom
[2]Department of Biomedical Sciences, University of Padova, via Ugo Bassi 58/B, Padova, 35131, Italy
[3]Istituto di Scienze e Tecnologie Chimiche ‘G. Natta’ SCITEC, Consiglio Nazionale delle Ricerche, via Mario Bianco 9 20131 Milano, Italy
[4]Human Technopole, Via Rita Levi Montalcini 1, 20157, Milano, Italy

[#]these authors contributed equally to this work
[⊥]current address: ELIXIR Hub, South Building, Wellcome Genome Campus, Hinxton, Cambridge, CB10 1SD, UK
[*]correspondence should be addressed to joseph.atherton@kcl.ac.uk or roberto.steiner@kcl.ac.uk or roberto.steiner@unipd.it

Supplementary Table 1. Cryo-EM data collection, refinement and validation statistics

	MT-Kif5b^{MoNeIAK}- -AMPPNP one-headed class (EMD-19174, PDB 8RHB)	MT-Kif5b^{MoNeIAK}- -AMPPNP two-headed class (EMD-19176, PDB 8RHH)	MT-Kif5b^{MoNeIAK}- -ADP one-headed class (EMD-19188 PDB 8RIK)	MT-Kif5b^{MoNeIAK}- -ADP two-headed class (EMD-19192 PDB 8RIZ)
**Data collection and processing**				
Magnification (actual)	46,168	46,168	46,168	46,168
Voltage (kV)	300kV	300kV	300kV	300kV
Electron exposure ($e^{-}/Å^2$)	52	52	52	52
Defocus range (µm)	-0.7 to -2.5µm	-0.7 to -2.5µm	-0.7 to -2.5µm	-0.7 to -2.5µm
Pixel size (Å)	1.083 Å	1.083 Å	1.083 Å	1.083 Å
Symmetry imposed*	Pseudo-helical	Pseudo-helical	Pseudo-helical	Pseudo-helical
Initial micrographs (#)	10,128	10,128	7,425	7,425
Initial MT segment particles[a] (#)	116,510	116,510	40,669	40,669
13pf MT segment particles[a] (#)	98,840	98,840	22,549	22,549
Final single particles used[a] (#)	633,952	570,836	110,731	39,512
Map resolution (Å)[b]	3.0 Å	3.0 Å, 3.4 Å	3.1 Å	3.6 Å, 3.9 Å
FSC threshold[c]	0.143	0.143	0.143	0.143
Map resolution range (Å)[b]	2.9-3.6 Å	2.9-5.6 Å, 3.5-4.1 Å	2.9-4.1 Å	3.3-9.3 Å, 3.6-7.8 Å
**Refinement**				
Initial models used	5SYF, 4HNA, 2Y65	5SYF, 4HNA, 2Y65, 1VFV	5SYF, 4LNU, 2Y65	5SYF, 4LNU, 2Y65, 1BG2
Refinement resolution (Å)	3.0	3.0	3.1	3.6
Model composition				
Nonhydrogen atoms	9,495	12,195	9,332	11,828
Protein residues	1,180	1,508	1,157	1,466
Ligands	6	8	5	7
R.m.s. deviations				
Bond lengths (Å)	0.003	0.003	0.003	0.002
Bond angles (°)	0.66	0.71	0.54	0.54
Validation[d]				
MolProbity score	1.58	1.63	1.48	1.53
Clashscore	7.6	8.09	4.72	7.78
Poor rotamers (%)	0	0.22	0	0.15
Ramachandran plot[d]				
Favored (%)	97.03	96.88	96.46	97.48
Allowed (%)	2.97	3.12	3.54	2.52
Disallowed (%)	0	0	0	0

(continues on the next page)

	MT-Kif5b^{MoNeIAK}- - AMPPNP all data	MT-Kif5b^{MoNeIAK}- - ADP all data	MT-Kif5b^{MoNeXXX}- - ADP (EMD-51477, PDB 9GNQ)
**Data collection and processing**			
Magnification	46,168	46,168	46,168
Voltage (kV)	300Kv	300Kv	300Kv
Electron exposure ($e^-/Å^2$)	52	52	52
Defocus range (μm)	-0.7 to -2.5μm	-0.7 to -2.5μm	-0.7 to -2.5μm
Pixel size (Å)	1.083Å	1.083Å	1.083 Å
Symmetry imposed*	Pseudo-helical	Pseudo-helical	Pseudo-helical
Initial micrographs (#)	10,128	7,425	4,884
Initial MT segment particles[a], (#)	116,510	40,669	125,234
13pf MT segment particles[a] (#)	94,840	22,594	89,119
Final single particles used[a] (#)	1,204,788	150,243	268,199
Map resolution (Å)[b]	2.9 Å	3.0 Å	2.9 Å
FSC threshold[c]	0.143	0.143	0.143
Map resolution range (Å)[b]	2.8-3.4 Å	2.9-4.1 Å	2.8-3.6 Å
**Refinement**			
Initial models used	n/a	n/a	5SYF, 4LNU, 2Y65
Refinement resolution (Å)	n/a	n/a	2.9 Å
Model composition			
Nonhydrogen atoms	n/a	n/a	9,309
Protein residues	n/a	n/a	1,156
Ligands	n/a	n/a	5
R.m.s. deviations			
Bond lengths (Å)	n/a	n/a	0.005
Bond angles (°)	n/a	n/a	0.58
Validation[d]			
MolProbity score	n/a	n/a	1.42
Clashscore	n/a	n/a	5.07
Poor rotamers (%)	n/a	n/a	0.99
Ramachandran plot[d]			
Favored (%)	n/a	n/a	97.15
Allowed (%)	n/a	n/a	2.76
Disallowed (%)	n/a	n/a	0.09

[a]MTs exhibit pseudo-helical symmetry due to a symmetry break at the seam. 13pf pseudo-helical MT segments were selected and processed, then single particles for each unique asymmetric unit produced using symmetry expansion (see methods).

[b]Two-headed classes were refined using either a mask inclusive of both kinesin heads and tubulin (1st stated range), or both kinesin heads alone (2nd stated range).

[c]The FSC curves for all reconstructions were subjected to the gold-standard noise-substitution test for overfitting[1], implemented in Relion. The resolution value at the noise-substitution test-corrected FSC 0.143 criterion is shown.

[d]As defined by the MolProbity[2] validation server.

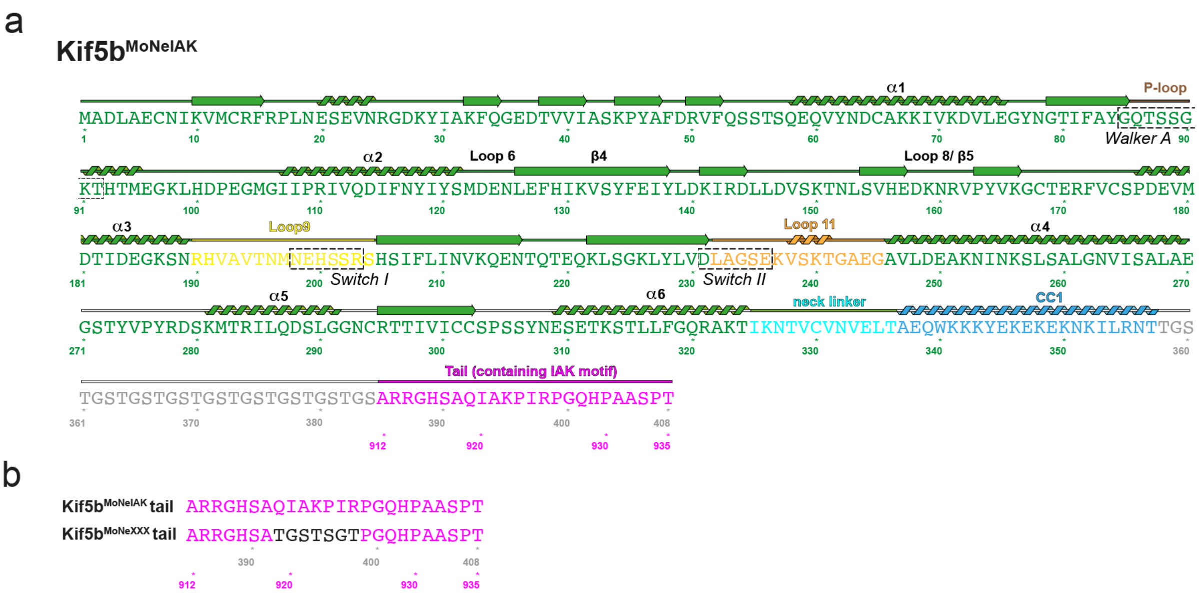

Supplementary Fig. 1. Kif5b^{MoNeIAK} and Kif5b^{MoNeXXX} chimeras and color scheme. a. Schematic of the Kif5b^{MoNeIAK} construct showing the motor domain's sequence, secondary structure elements (SSE), motifs, and coloring scheme used in figures. SSE annotation is based on the structure of our MT-bound structure in the presence of AMPPNP. **b**. Sequence alignment of the Kif5b^{MoNeIAK} and Kif5b^{MoNeXXX} tail regions, (sequence numbering as in panel **a**), with Kif5b^{MoNeXXX} IAK-motif substitutions shown in black.

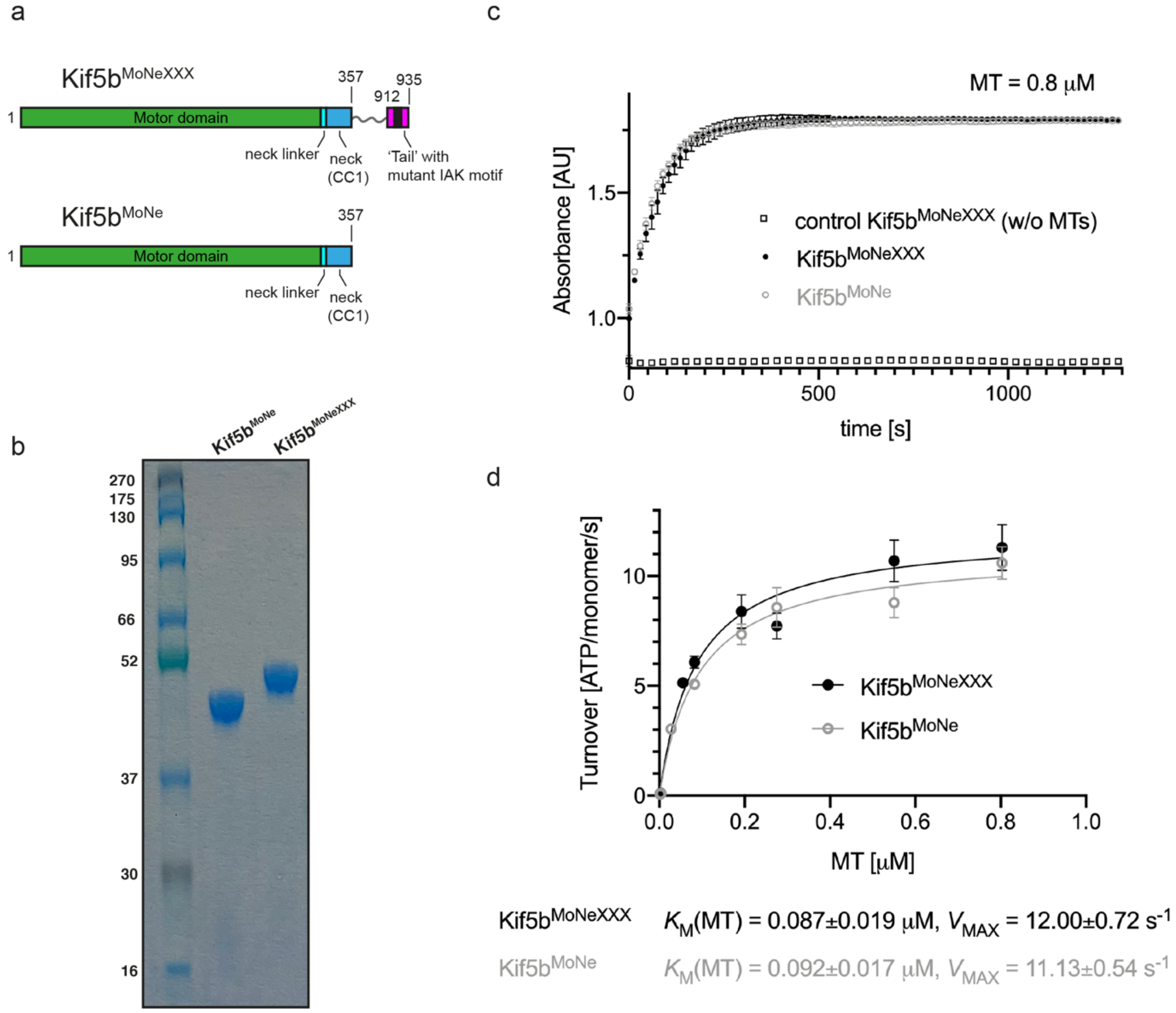

Supplementary Fig. 2. ATPase assay to validate the chimeric approach. a. Schematics of the Kif5b^{MoNeXXX} chimera (identical to that shown in Fig. 1a of the main text) and of the isolated motor without the engineered extension (Kif5b^{MoNe}). The latter corresponds to Kif5b(1-357). **b**. SDS-PAGE of purified His$_6$-Kif5b^{MoNeXXX} and His$_6$-Kif5b^{MoNe} used for the ATPase assay. **c**. Representative time course of ATPase activity at fixed MT concentration. Both His$_6$-Kif5b^{MoNeXXX} are His$_6$-Kif5b^{MoNe} activated by MTs. In a control experiment (His$_6$-Kif5b^{MoNeXXX} only) there is no detectable ATPase activity in the absence of MTs. Data points are mean of three replicates. Error bars are s.e.m. values. **d**. Turnover as function of MT concentration. Maximal turnover, V_{MAX} and apparent Michaelis-Menten values, K_M(MT) were determined by fitting the Michaelis-Menten equation using the Prism package (version 8.3.1(332) for macOS, GraphPad Software). Kinetic parameters are identical within error. Data points are mean of three replicates. Error bars are s.e.m. values. Source data for panels (**c**,**d**) are provided as a Source Data file.

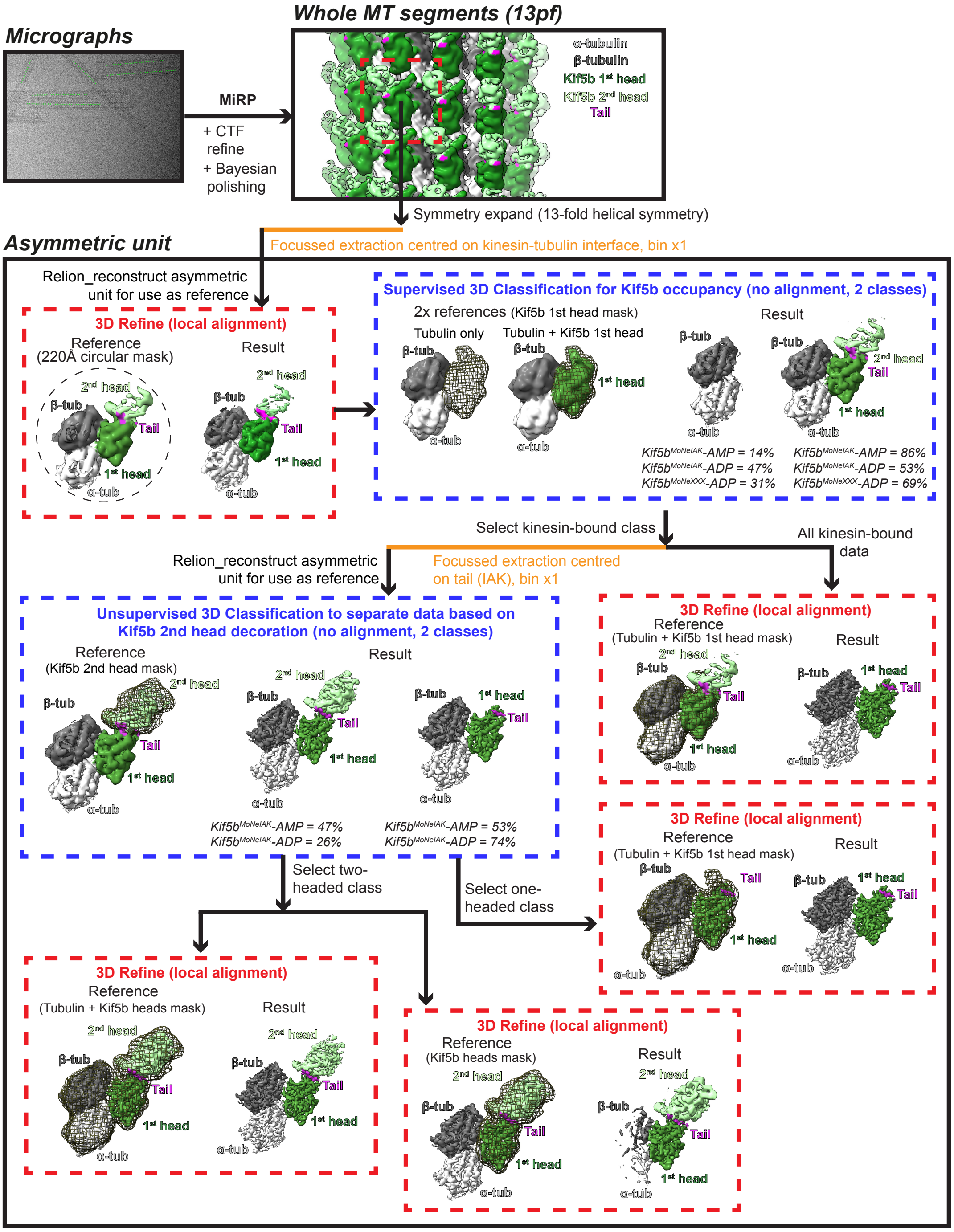

Supplementary Fig. 3. Schematic showing the processing strategy for MT-associated Kif5bMoNeIAKand Kif5bMoNeXXX. A full explanation is given in the Methods section, but in brief, micrographs were picked manually in Relion and then whole MT segments processed with MiRP3running in Relionv3.0. Symmetry expansion and focused extraction then allowed us to perform focused refinements and classifications on different portions of the asymmetric unit in using smaller, less memory-intensive box sizes. Focused refinement steps are shown in red, focused extraction steps in shown in orange, focused classification steps shown in blue and other processing steps shown in black. Class occupancies for the various datasets are indicated below the classes. Mesh density indicates a 3D reference mask. Local refinements included a translational search radius of ±5 pixels and an angular search range of ±1.5°. All cryo-EM density shown is unfiltered.

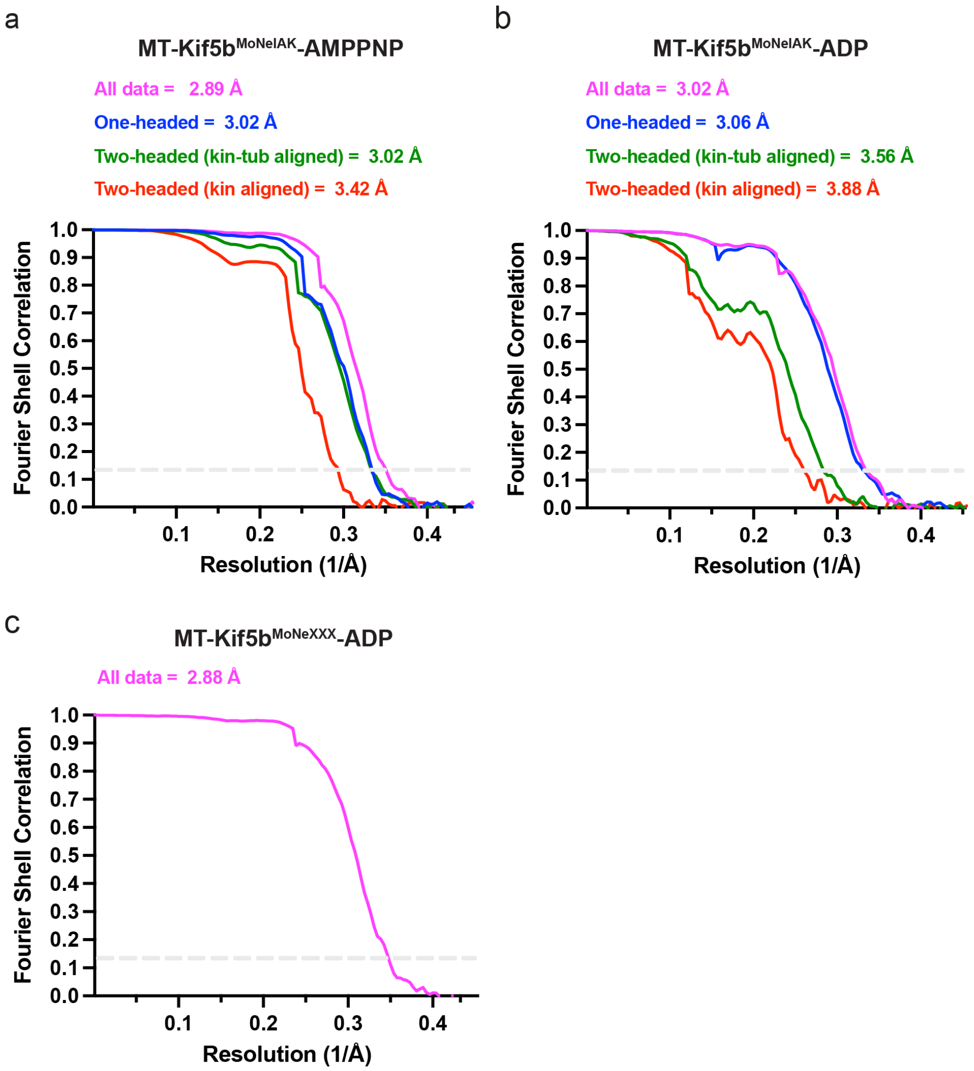

Supplementary Fig. 4. Global FSC curves for all reconstructions. a,b,c. Noise-substitution test-corrected FSC curves (derived from Relion v3.0) are shown for focused asymmetric unit reconstructions from (**a**) MT-Kif5b^{MoNeIAK}-AMPPNP, (**b**) MT-Kif5b^{MoNeIAK}-ADP and (**c**) MT-Kif5b^{MoNeXXX}-ADP datasets. For all datasets, reconstructions were either of: *i*) all data refined using a tubulin plus kinesin motor domain-inclusive mask (magenta); *ii*) the one-headed state refined using a tubulin+kinesin motor domain-inclusive mask (blue); the two-headed state refined using either *iii*) a tubulin+both kinesin motor domains-inclusive mask (green) or *iv*) both kinesin motor domains-inclusive mask (excluding tubulin, red). Soft loose masks were used in focused refinements and the same masks used in FSC calculations. Global resolutions in Å are reported at the gold-standard 0.143 FSC cut-off criterion for the noise-substitution test-corrected FSC curves.

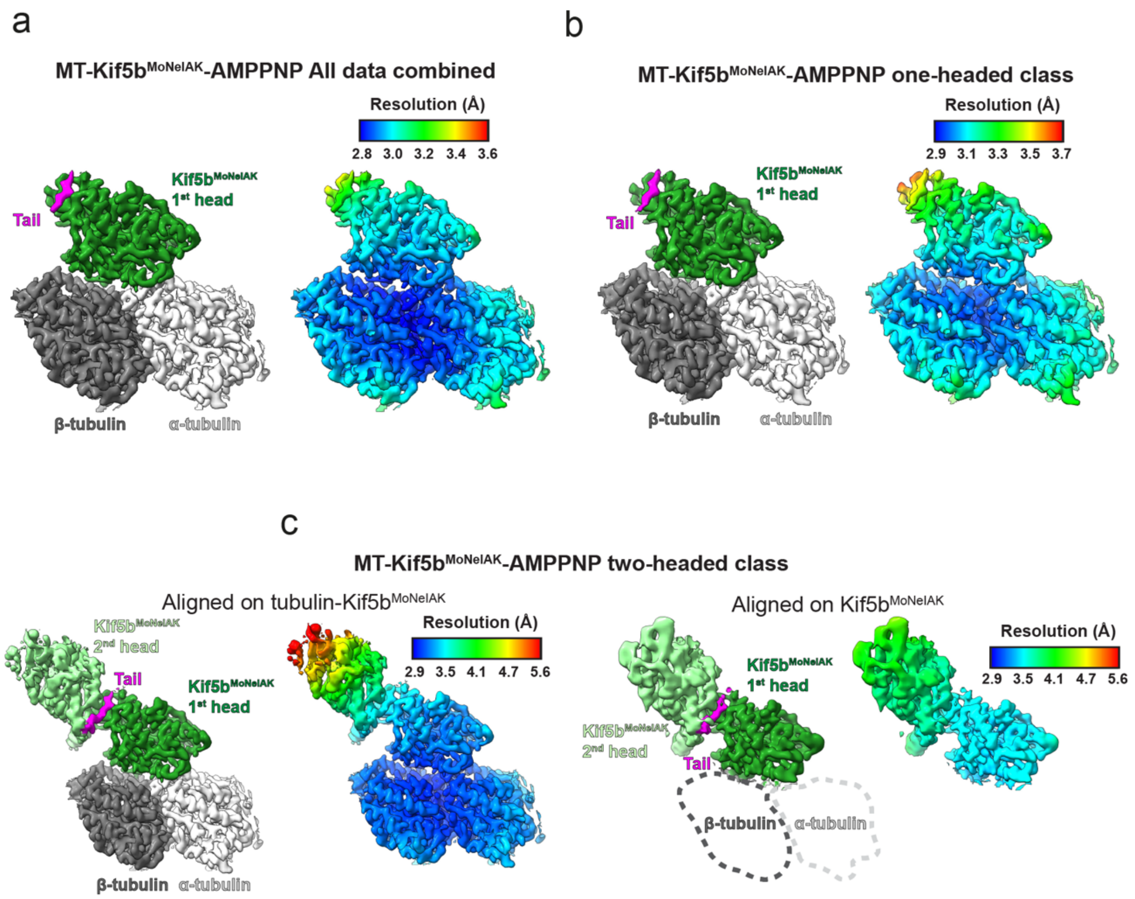

Supplementary Fig. 5. Local resolution estimates for MT-Kif5b^{MoNeIAK}-AMPPNP reconstructions. a,b,c. Density for asymmetric units is colored either according to segmentation into kinesin motor domain, tubulin and tail densities (left-hand panels) or local resolution (right-hand panels). Similar views are shown for (**a**) all data refined using a tubulin plus kinesin motor domain-inclusive mask, (**b**) the one-headed state refined using a tubulin+kinesin motor domain-inclusive mask and (**c**) the two-headed state refined using either a tubulin+both kinesin motor domains-inclusive mask (left hand panels) or both kinesin motor domains-inclusive mask excluding tubulin whose location is indicated by dashed shapes (right hand panels). Unfiltered/unsharpened final reconstructions are shown with density zoned according to focused masks used for refinement. Local resolutions were estimated with Relion v3.0's inbuilt software. Color keys are provided above the local-resolution density depictions. All cryo-EM density shown is unfiltered.

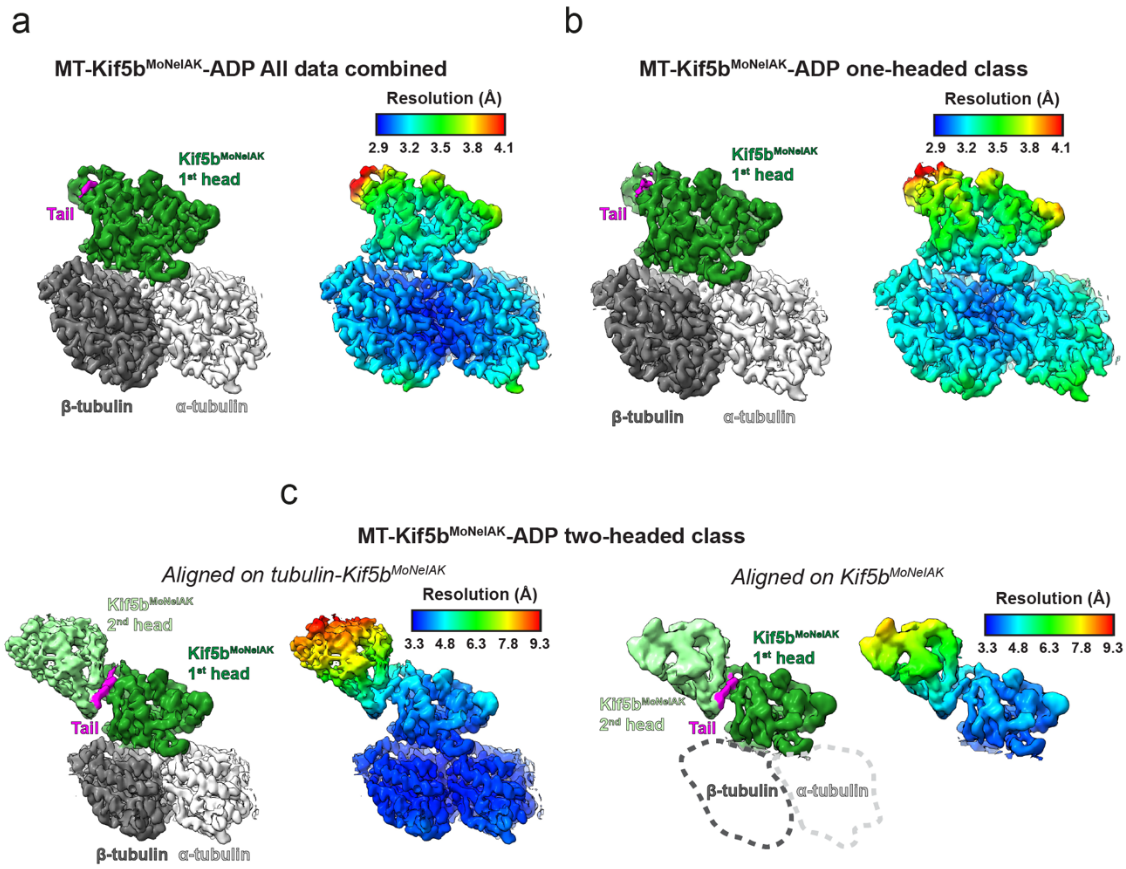

Supplementary Fig. 6. Local resolution estimates for MT-Kif5b^{MoNeIAK}-ADP reconstructions. **a,b,c.** Density for asymmetric units is colored either according to segmentation into kinesin motor domain, tubulin and tail densities (left-hand panels) or local resolution (right-hand panels). Similar views are shown for (**a**) all data refined using a tubulin plus kinesin motor domain-inclusive mask, (**b**) the one-headed state refined using a tubulin+kinesin motor domain-inclusive mask and (**c**) the two-headed state refined using either a tubulin+both kinesin motor domains-inclusive mask (left hand panels) or both kinesin motor domains-inclusive mask excluding tubulin which is indicated by dashed shapes (right hand panels). Unfiltered/unsharpened final reconstructions are shown with density zoned according to focused masks used for refinement. Local resolutions were estimated with Relion v3.0's inbuilt software. Color keys are provided above the local-resolution density depictions. All cryo-EM density shown is unfiltered.

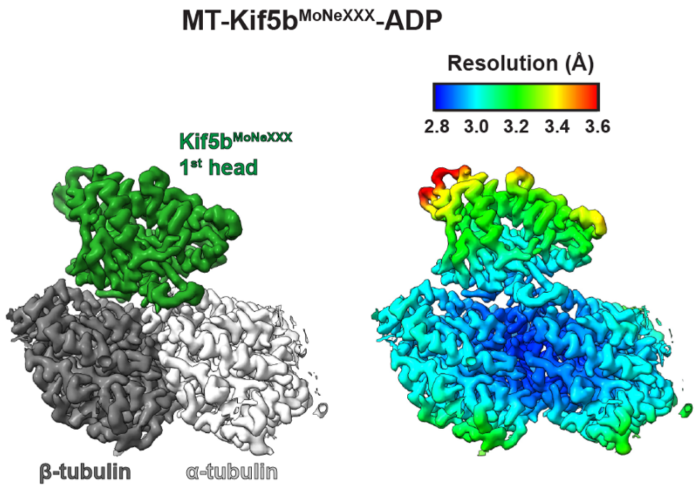

Supplementary Fig. 7. Local resolution estimates for the MT-Kif5b^{MoNeXXX}-ADP reconstruction. Density for the asymmetric unit is colored either according to segmentation into kinesin motor domain, tubulin and tail densities (left-hand panel) or local resolution (right-hand panel). The unfiltered/unsharpened final reconstruction is shown with density zoned according to focused masks used for refinement. Local resolution was estimated with Relion v3.0's inbuilt software. A color key is provided above the local-resolution density depictions. All cryo-EM density shown is unfiltered.

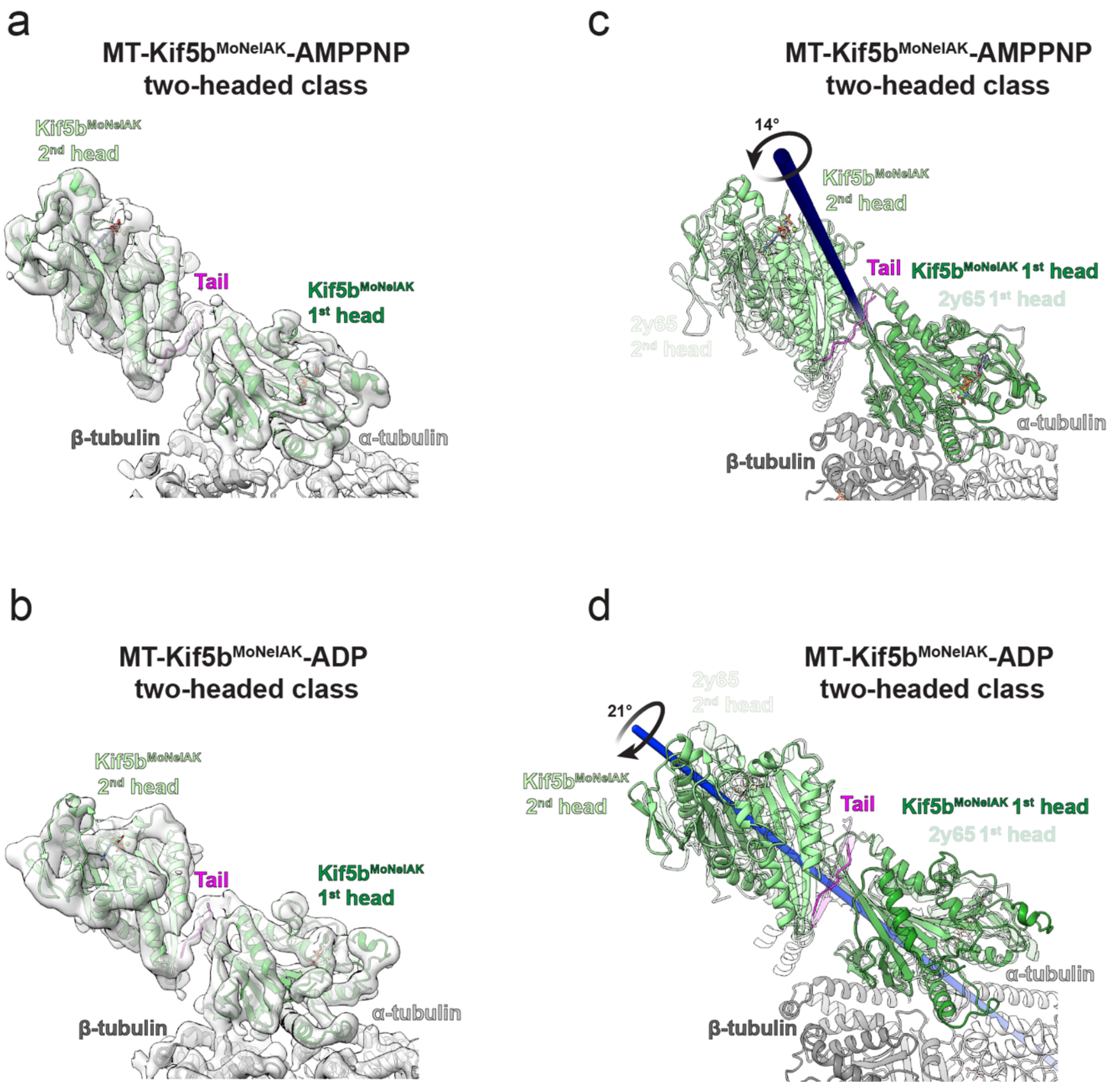

Supplementary Fig. 8. The orientation of the 2nd head relative to the 1st is altered compared to the crystal structure of *Drosophila* kinesin-1 dimer + tail. a,b. MT-Kif5b^{MoNeIAK} two-headed models bound to (**a**) AMPPNP or (**b**) ADP are shown in their respective cryo-EM densities (transparent grey) low-pass filtered to 6 Å (appropriate for visualization of the whole complex). **c,d.** Superimpositions of the X-ray crystallographic structure of *Drosophila melanogaster* kinesin-1 in complex with a tail peptide (semi-transparent, PDB code 2y65)[3] onto loop 8 and β4 (tail-binding regions) of the MT-associated head of the Kif5b^{MoNeIAK} two-headed models (opaque) bound to either (**c**) AMPPNP (top panel) or (**d**) ADP. The rotation axes (blue) and angles of the 2nd Kif5b^{MoNeIAK} head relative to the 2nd head of the crystallographic model are shown.

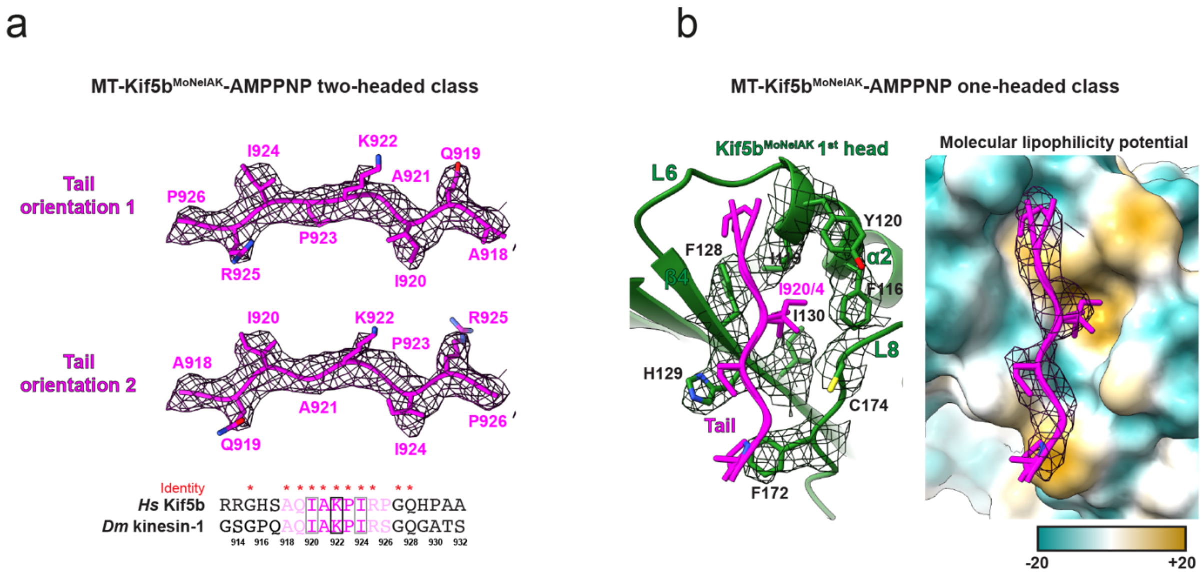

Supplementary Fig. 9. Binding site and mixed polarity of the tail IAK-motif. a. Tail density for the two-headed class of MT-Kif5b^{MoNeIAK} in the presence of AMPPNP with two modelled orientations of the pseudo-palindromic tail sequence with amino acid numberings. A sequence alignment of the IAK-motif containing tail region of human Kif5b (*Hs*Kif5B) and *Drosophila melanogaster* kinesin-1 (*Dm*kinesin-1) is shown below, with identical residues illustrated with red asterisks. Light and solid magenta sequence coloring shows residues resolved in the presence of AMPPNP, with residues only resolved in the presence of ADP shown in solid magenta. The central lysine K922 of the pseudo-palindrome is boxed in black, whereas key isoleucine residues I920 and I924 either side are boxed in grey. **b.** The motor domain head (green) binding site of the tail (magenta) is shown on the AMPPNP-bound one-headed state, with focus on the embedding of I920/4 a hydrophobic pocket. The left-hand panel shows the motor domain model binding site, with side chains and density (mesh) for key Kif5b hydrophobic pocket residues associated with the tail. The right-hand panel shows the same view, but with tail density shown in mesh and the motor domain displayed as a surface colored by hydrophobicity (molecular lipophilicity potential calculated by ChimeraX[4], range -20 to +20). Cryo-EM density shown was filtered using LocScale[6].

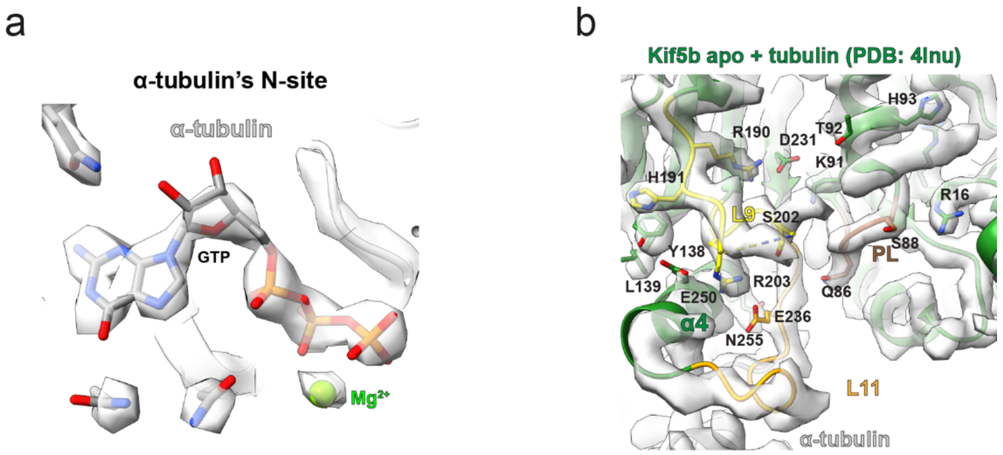

Supplementary Fig. 10. The MT- and ADP-bound Kif5b^{MoNeXXX} complex is apo-like and retains Mg^{2+} in α-tubulin's N-site. a. α-tubulin's non-exchangeable GTP binding site in the reconstruction from all MT-associated Kif5b^{MoNeXXX}-ADP data, showing clear cryo-EM density (semi-transparent grey) for a Mg^{2+} ion associated with GTP. **b.** Overview of the nucleotide pocket and switch-motif containing loops L9 and L11 in the nucleotide-free (apo) structure of Kif5b motor domain on tubulin (PDB code 4lnu)[5], with side chains for key conserved residues shown. The model is fitted into the cryo-EM density for MT-associated Kif5b^{MoNeXXX}-ADP (semi-transparent), showing the high structural similarity (apart from the missing ADP). Cryo-EM density displayed in panel (**a**) or (**b**) was produced using Relion's local resolution filtering tool and DeepEMhancer[8], respectively.

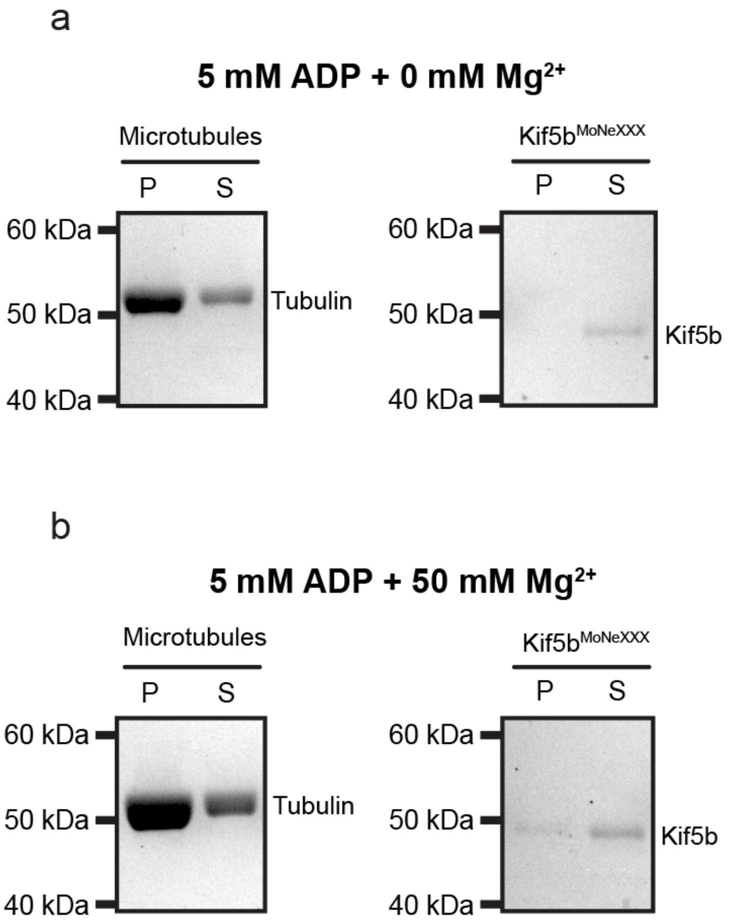

Supplementary Fig. 11. Controls for the co-sedimentation assay shown in Fig. 3c,d of the main text. a,b. Controls of MT-only or Kif5b^{MoNeXXX} only samples sedimentation with either (**a**) no $MgCl_2$ or (**b**) 50 mM $MgCl_2$. The experiment was performed in BRB80 buffer with 5 mM ADP and the indicated concentrations of $MgCl_2$, with taxol-stabilized MTs at 2 µM (tubulin dimer) and 0.5 µM Kif5b^{MoNeXXX}. For conditions without Mg^{2+} we used 10 mM EDTA to remove any possible free ion present. P = Pellet, S = Supernatant.

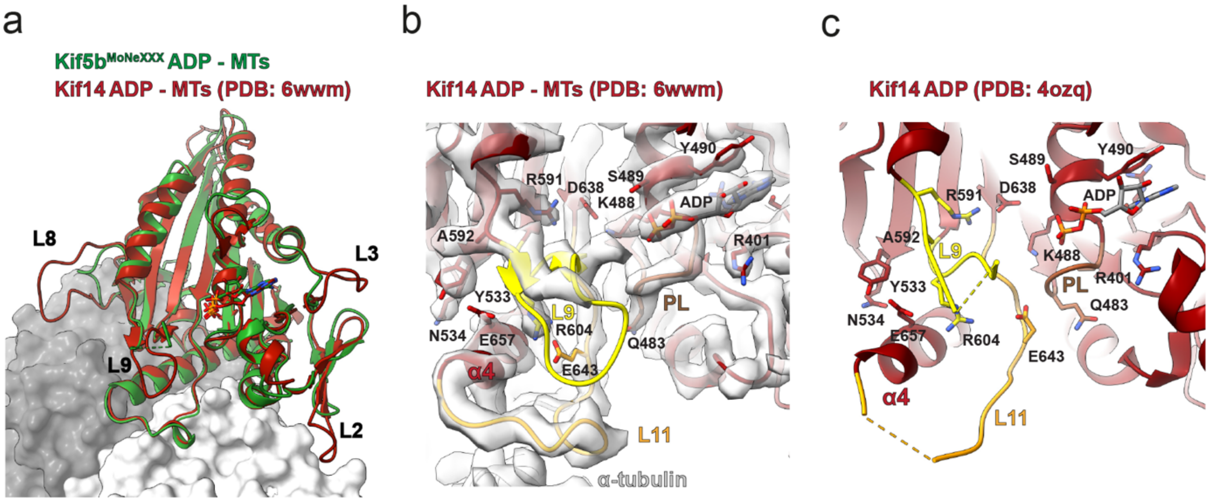

Supplementary Fig. 12. Comparison of Kif14 apo-like ADP states with Kif5b^{MoNeXXX}-ADP structure. a. ADP-bound motor domains of the Kif5b^{MoNeXXX} (green) and Kif14 (maroon, PDB code 6wwm)[6] MT-associated models are superimposed, with divergent loops labelled. The tubulin dimer from the Kif5b^{MoNeXXX} asymmetric unit is shown as a grey surface representation. **b**. Overview of the kinesin nucleotide-pocket and switch-motif containing loops L9 and L11, where the Kif14-ADP MT-associated model is fitted into our microtubule-associated Kif5b^{MoNeXXX}-ADP cryo-EM density (semi-transparent), showing high similarity apart from L9's apex and certain divergent amino acid side chains. Cryo-EM density was filtered using DeepEMhancer[8]. **c.** A similar view to panel (**b**), but instead showing the structure of Kif14-ADP in the absence of MTs (PDB code 4ozq)[7] without cryo-EM density. Sequence numberings for Kif14 are shown.

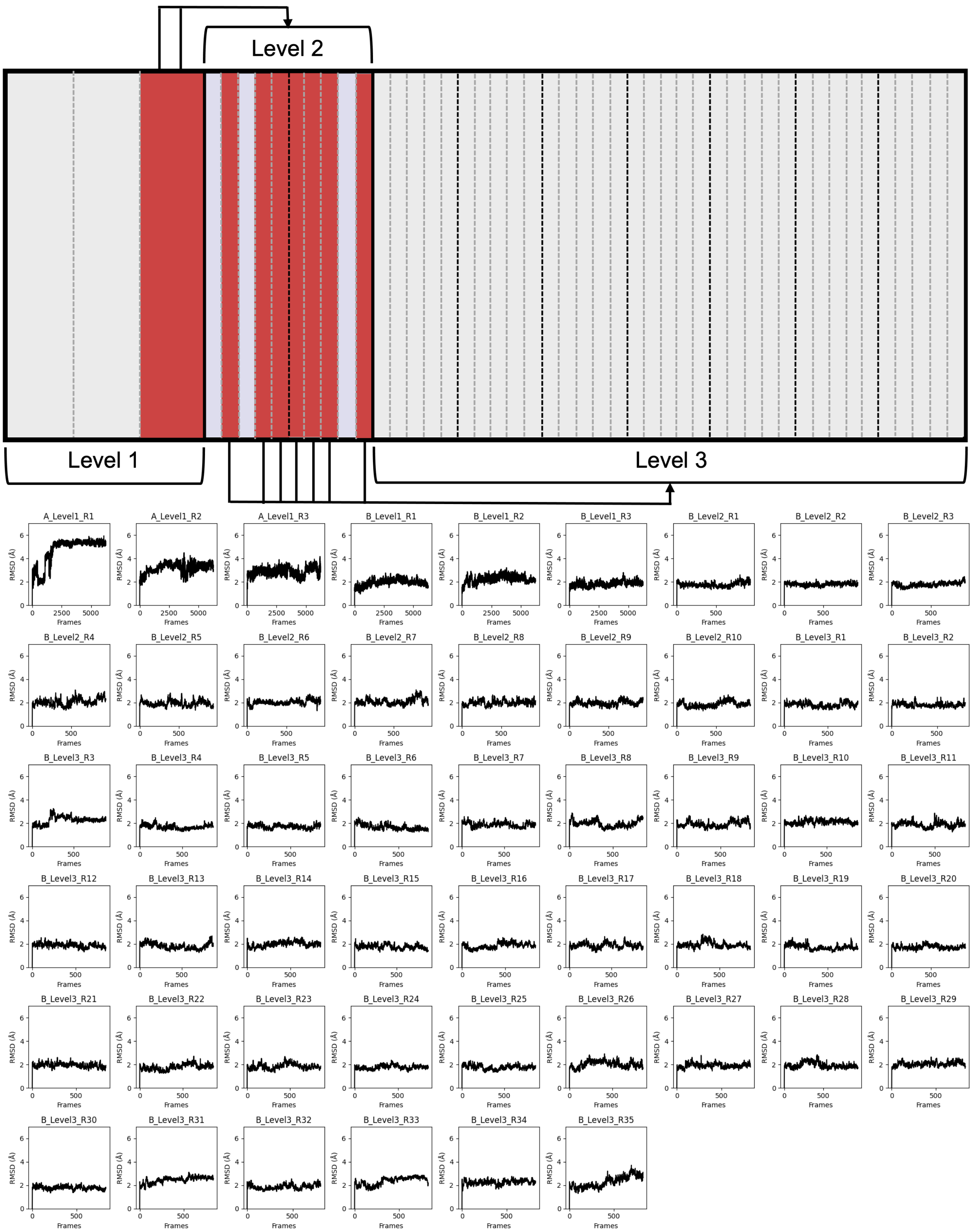

Supplementary Fig. 13. Molecular Dynamics scheme applied to the MT-bound system. **a.** diagram showing the trajectory scheme applied to MT-bound/Mg^{2+}-free system (see main text). Each vertical bar represents an independent replica. Level 1 consists of 3 replicas of 1μs each from the starting $Kif5b^{MoNeXXX}$ model. Level 2 is obtained by running 5 replicas of 150 ns from each one of two snapshots extracted from replica 3 on Level 1 (highlighted in red). Level 3 consists of 5 replicas of 150 ns started from each one of 7 snapshots, extracted from a subset of trajectories on Level 2 (highlighted in red). **b.** timeline plots showing the RMSD of motor domain Cα atoms for the full set of simulations presented in this study (A = MT-free/Mg^{2+}-bound system, B = MT-bound/Mg^{2+}-free system, R# = replica number).

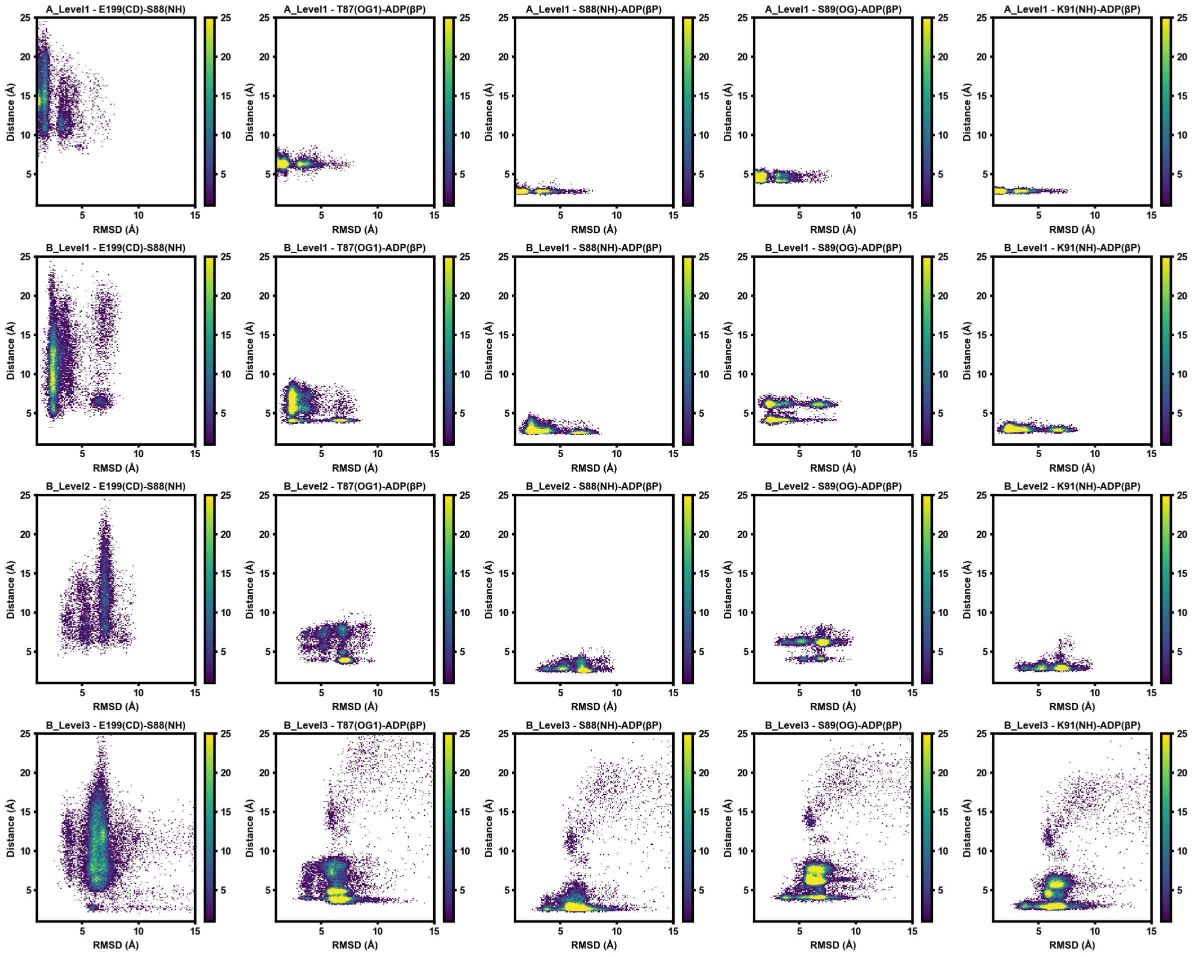

Supplementary Fig. 14. Distribution of the set of reaction coordinates in the full MD dataset. 2D histograms (frequency on the right-hand sidebar) showing the population of pair distances E199(CD)-S88(NH), T87(OG1)-ADP(βP), S88(NH)-ADP(βP), S89(OG)-ADP(βP) and K91(NH)-ADP(βP) versus the RMSD ADP during the MD runs. A = MT-free/Mg^{2+}-bound system, B = MT-bound/Mg^{2+}-free system. Levels are as defined in Supplementary Fig. 13 and in the Methods.

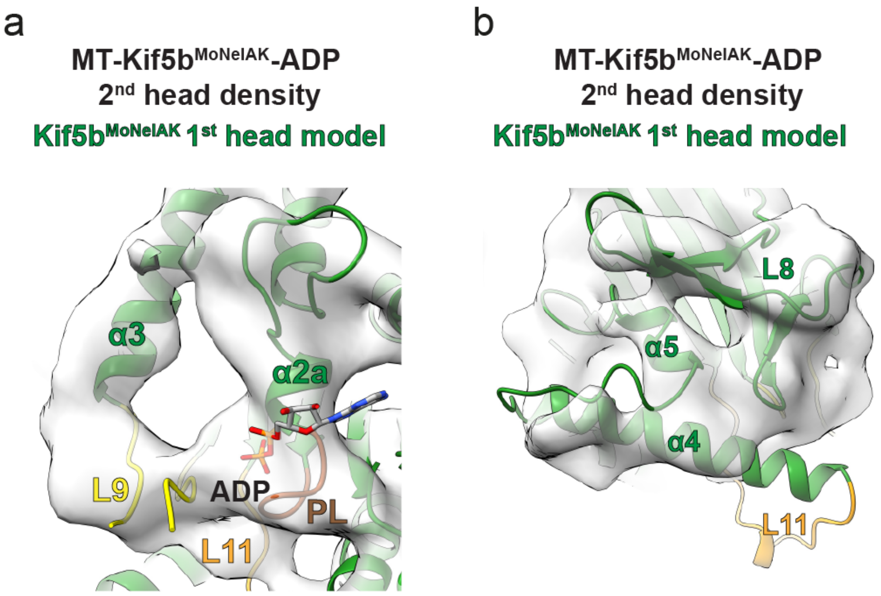

Supplementary Fig. 15. In the presence of ADP, the 2nd head of the Kif5b^{MoNeIAK} two-headed state adopts a distinct conformation compared to the 1st head. Cryo-EM density (semi-transparent, low-pass filtered to 6 Å) is shown for the 2nd (not MT-associated) Kif5b^{MoNeIAK} head in the presence of ADP with views of (**a**) the nucleotide pocket and (**b**), the MT binding elements (including helix-α4 and L11) and the neck linker. The model for the Kif5b^{MoNeIAK} microtubule-associated 1st head is fitted into the density for the 2nd head, showing the two heads adopt distinct conformations (compare with Fig. 5f,g).

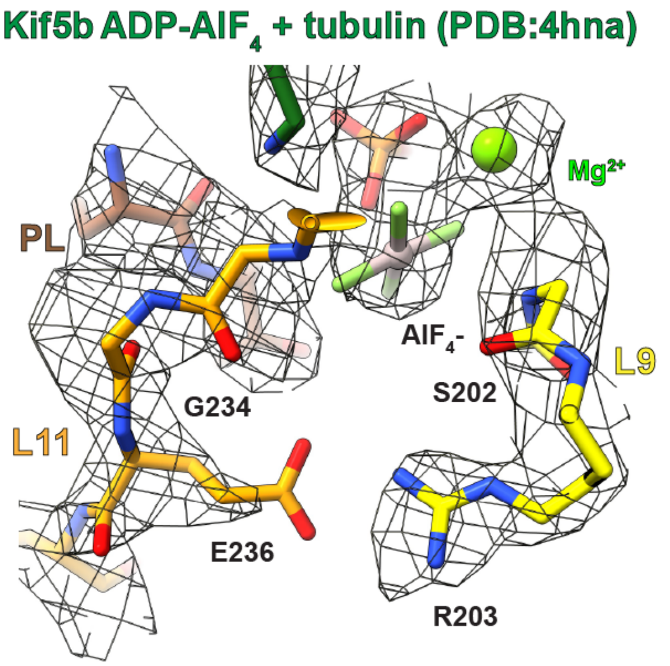

Supplementary Fig. 16. The ATP-like state of nucleotide pocket is indistinguishable in tail-bound and tailless Kif5b. Close up view of residues involved in ATP hydrolysis, with the tubulin and ADP-AlF_4-bound tailless crystal structure of the Kif5b motor domain (PDB code 4hna)[8] fitted into density for the MT-associated motor domain of AMPPNP-bound Kif5b^{MoNeIAK} (mesh). Cryo-EM density was filtered using Relion's local resolution sharpening tool.

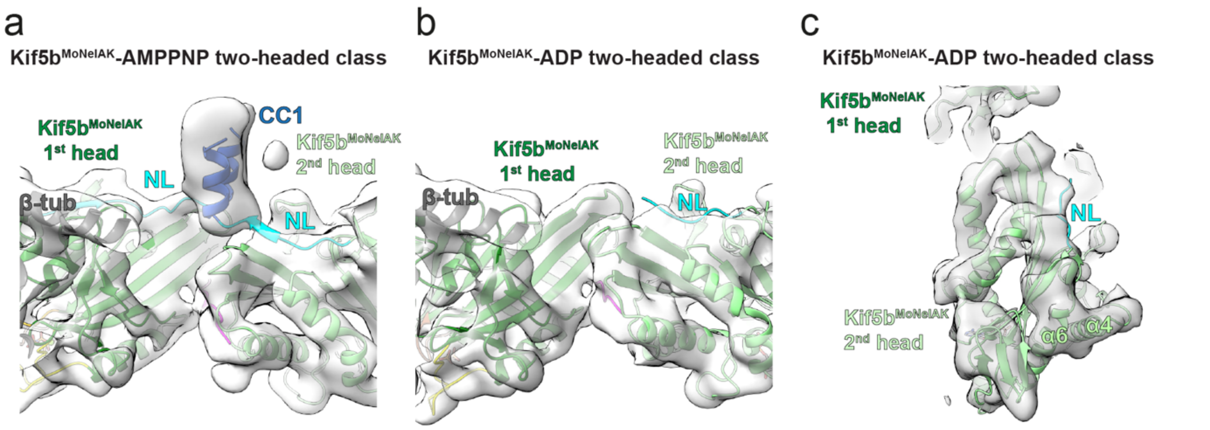

Supplementary Fig. 17. Neck linkers of both heads in the two-headed state only dock in the presence of AMPPNP, such that CC1 is observed. a,**b.** Views of the inter-head interface of the Kif5b^{MoNeIAK} two-headed state in the presence of (**a**) AMPPNP or (**b**) ADP, with the corresponding semi-transparent density (low-pass filtered to 6 Å, appropriate for visualization of the whole complex) and fitted models shown. Both neck-linkers (NL) are only docked in the presence of AMPPNP, such that CC1 is stabilized. **c.** Alternative view of the 2nd head in the ADP-bound Kif5b^{MoNeIAK} two-headed state, showing docking of the neck linker.

References

1. Chen, S. *et al.* High-resolution noise substitution to measure overfitting and validate resolution in 3D structure determination by single particle electron cryomicroscopy. *Ultramicroscopy* **135**, 24-35 (2013). https://doi.org:10.1016/j.ultramic.2013.06.004
2. Williams, C. J. *et al.* MolProbity: More and better reference data for improved all-atom structure validation. *Protein Sci* **27**, 293-315 (2018). https://doi.org:10.1002/pro.3330
3. Kaan, H. Y., Hackney, D. D. & Kozielski, F. The structure of the kinesin-1 motor-tail complex reveals the mechanism of autoinhibition. *Science* **333**, 883-885 (2011). https://doi.org:10.1126/science.1204824
4. Meng, E. C. *et al.* UCSF ChimeraX: Tools for structure building and analysis. *Protein Sci* **32**, e4792 (2023). https://doi.org:10.1002/pro.4792
5. Cao, L. *et al.* The structure of apo-kinesin bound to tubulin links the nucleotide cycle to movement. *Nat Commun* **5**, 5364 (2014). https://doi.org:10.1038/ncomms6364
6. Benoit, M. *et al.* Structural basis of mechano-chemical coupling by the mitotic kinesin KIF14. *Nat Commun* **12**, 3637 (2021). https://doi.org:10.1038/s41467-021-23581-3
7. Arora, K. *et al.* KIF14 binds tightly to microtubules and adopts a rigor-like conformation. *J Mol Biol* **426**, 2997-3015 (2014). https://doi.org:10.1016/j.jmb.2014.05.030
8. Gigant, B. *et al.* Structure of a kinesin-tubulin complex and implications for kinesin motility. *Nat Struct Mol Biol* **20**, 1001-1007 (2013). https://doi.org:10.1038/nsmb.2624

Uncropped SDS-PAGE gels

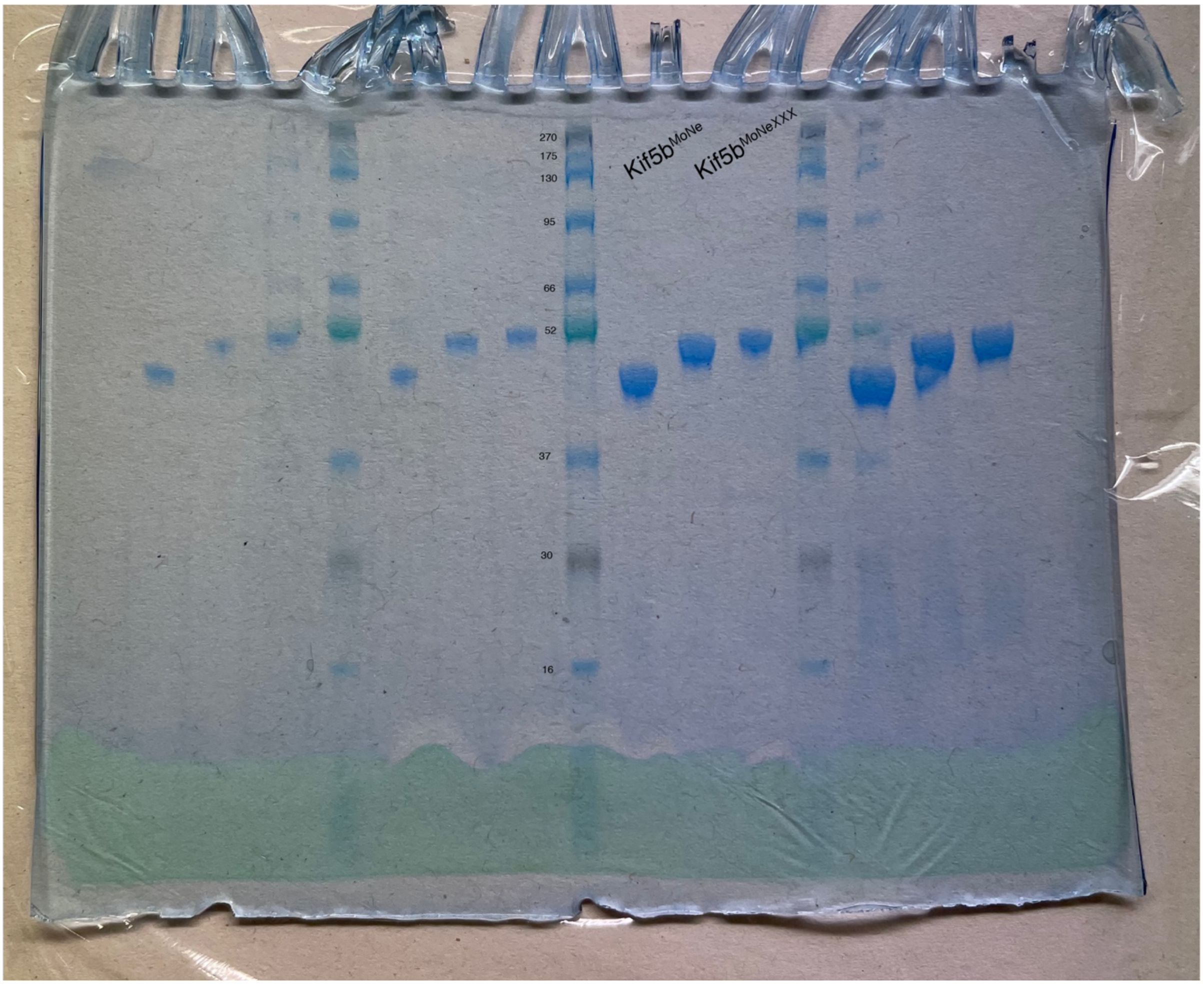

Supplementary Fig. 2b.

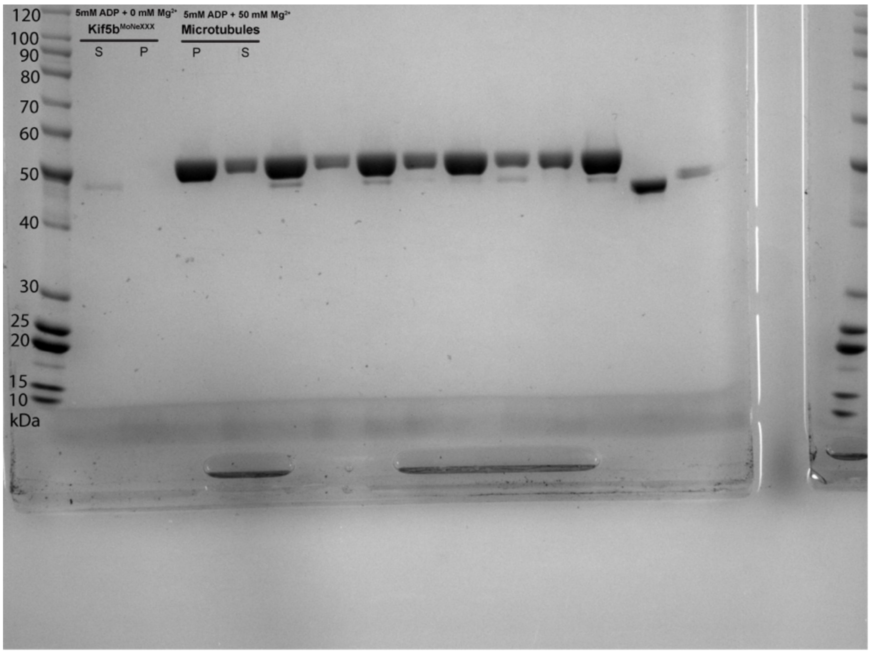

Supplementary Fig. 11a(right) and 11b(left).

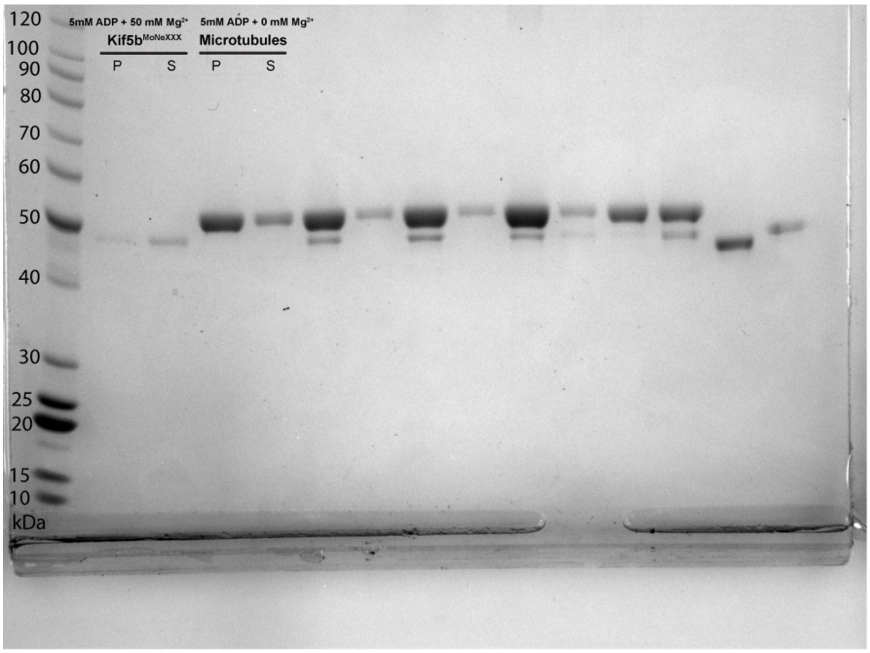

Supplementary Fig. 11a(left) and 11b(right).
